# Glycosaminoglycan, Antimicrobial Defence Molecule and Cytokine Appearance in Tracheal Hyaline Cartilage of Healthy Humans

**DOI:** 10.3390/jfmk7030055

**Published:** 2022-07-21

**Authors:** Arina Deņisova, Māra Pilmane, Pavlo Fedirko

**Affiliations:** 1 Institute of Anatomy and Anthropology, Riga Stradins University, LV-1010 Riga, Latvia; mara.pilmane@rsu.lv; 2 Institute of Radiation Hygiene and Epidemiology, State Institution “National Research Center for Radiation Medicine of the National Academy of Medical Sciences of Ukraine”, 04050 Kyiv, Ukraine; rad_epid@ukr.net

**Keywords:** trachea, hyaline cartilage, glycosaminoglycans, human beta defensins, interleukin 10, human cathelicidin

## Abstract

Hyaline cartilage is an important tracheal structure, yet little is known about its molecular composition, complicating investigation of pathologies and replacement options. Our aim was to research tracheal hyaline cartilage structure, protective tissue factors and variations in healthy humans. The tissue material was obtained from 10 cadavers obtained from the Riga Stradins University Institute of Anatomy and Anthropology archive. Tissues were stained with Bismarck brown and PAS for glycosaminoglycans, and immunohistochemistry was performed for HBD-2, HBD-3, HBD-4, IL-10 and LL-37. The slides were inspected by light microscopy and Spearman’s rank correlation coefficient was calculated. The extracellular matrix was positive across hyaline cartilage for PAS, yet Bismarck brown marked positive proliferation and growth zones. Numerous positive cells for both factors were found in all zones. All of the antimicrobial defence molecules and cytokines were found in a moderate number of cells, except in the mature cell zone with few positive cells. Spearman’s rank correlation coefficient revealed strong and moderate correlations between studied factors. Hyaline cartilage is a tracheal defence structure with a moderate number of antimicrobial defence protein and cytokine immunoreactive cells as well as numerous glycosaminoglycan positive cells. The extracellular matrix glycosaminoglycans provide structural scaffolding and intercellular signalling. The correlations between the studied factors confirm the synergistic activity of them.

## 1. Introduction

The trachea is an organ of the respiratory system, which consists of approximately 20 incomplete hyaline cartilage rings, smooth muscle, elastic connective tissue, respiratory epithelium as well as seromucous glands. Having the function of air conduction, conditioning and warming, as well as removal of inspired particles including dust, it is safe to say that the trachea is a necessary and very important part of the respiratory system [1]. However, because tracheal illness symptoms are frequently associated with other respiratory tract organs, such as the larynx or bronchi, it is often clinically overlooked and has become a blind spot in clinical practice [2]. Tracheal diseases are still present, and the majority of them are associated with iatrogenic causes or trauma. For example, 1–3% of intensive care unit patients undergoing ventilation develop tracheobronchitis and up to 21% of intubated patients, and specifically 32% of patients with tracheostomies, develop tracheal stenosis [2,3]. Moreover, a study that was conducted in Germany by Schibilsky et al. in 2020 showed that traumatic tracheobronchial injuries have a very high risk of mortality and mostly occur in males secondary to blunt trauma during accidents [4]. Tracheal impairment can also be caused by malignant tumors (squamous cell or adenoid cystic carcinoma and thyroid cancer), congenital stenosis, tracheoesophageal fistula and idiopathic causes [5,6].

Tracheal diseases lead not only to respiratory impairment, but also permanent disability, sometimes requiring tracheal resection or in some cases even tracheal replacement, in which hyaline cartilage is one of the most significant parts [5,7]. The problem arises with choosing an appropriate material and method for the replacement. Currently, the best results are achieved with autologous tissue composite; however, there is no method which could be a perfect fit for all patients and have no complications due to the complex structure of the trachea [5,8]. Although there are studies about possible tracheal replacement materials, more research related to the normal composition of the trachea, including structure and physiology of its hyaline cartilage, could possibly reveal the necessary information that could help in future therapeutic development [7].

Thus, despite particular knowledge in ground substance structure and glycosaminoglycan distribution, such knowledge regarding the appearance of antimicrobial defence molecules and cytokines is limited.

Tracheal hyaline cartilage makes up the structural support of the respiratory tract. The cells that are found in hyaline cartilage are mesenchymal cells that differentiate into chondroblasts that later make up chondrocytes. Although the cartilage itself is avascular and the necessary nutrients diffuse through the extracellular matrix (ECM) from perichondria during body movements, which is the reason why cartilage is the part of the trachea with poor regenerative capacity, its cells are still biologically active and secrete different molecules. The extracellular matrix which surrounds cartilaginous cells consists of collagen, mostly type 2, which is intertwined with very well hydrated proteoglycans, including aggrecans, that are composed of protein core and glycosaminoglycans (GAGs) [9,10].

Glycosaminoglycans are molecules, consisting of long and unbranched polysaccharide chains, containing repeated disaccharide units and are components of the extracellular matrix. The functions of GAG include cell hydration, providing structural scaffolding, as well as allowing cell signalling through modulation of different biochemical processes [11]. In hyaline cartilage, three different glycosaminoglycans can be found: chondroitin sulphate, keratan sulphate and hyaluronic acid [12,13,14]. In articular cartilage, chondroitin sulphate is territorial; however, keratan sulphate is inter-territorial, except in the deepest zones of tissues that are older, where keratan sulphate is localized closer to chondrocytes [15]. The natural process of aging has a huge impact on GAG composition in hyaline cartilage. A study conducted by Riedler et al. in 2017 showed that glycosaminoglycans in hyaline cartilage of the nasal septum decrease with age, together with chondrocyte density and cell size, and that this occurs even faster than in elastic cartilage found in the auricle [16].

Although the extracellular matrix of the hyaline cartilage is rich with nutrients and biologically active molecules that diffuse from the perichondrium which is rich in vascular supply, it is important to note that the cells of hyaline cartilage are also biologically active and secrete various molecules in response to different stimuli [9]. Besides natural GAG, particular interest is devoted to tissue protection factors such as antimicrobial proteins and some specific interleukins (IL), such as interleukin 10 (IL-10), as the intercorrelations of the above mentioned might be important for the circumstances related to the wellbeing of cartilages and other hard structures [17,18].

Human beta defensin 2 (HBD-2) is an antimicrobial peptide which belongs to the beta defensin family, which is mostly expressed in the epithelial cells of respiratory and digestive tracts as well as in skin and conjunctive epithelium in response to pro-inflammatory cytokines, such as IL-1α, IL-1β, TNF-α, IFN-γ, IL-17 and IL-22 [19,20]. Its main role is in providing immunity against pathogens. When the cationic HBD-2 molecule comes near the bacterial surface, an electrostatic attraction forms because of the negatively charged bacterial components. Subsequently, HBD-2 gets integrated in the membrane of the bacteria, where it later forms pores and makes the membrane permeable, thus inducing destruction of the microorganism [19,20]. Eukaryotic cells have more cholesterol in them, making the membrane neutral and thus HBD-2 does not pose a threat to them. HBD-2 also serves as a chemoattractant for dendritic cells and memory T cells through formation of antigen-defensin complexes and facilitates antigen recognition. However, it also has anti-inflammatory properties because of its inhibitory effects against the complement system, especially C1q, that allows the blockage of the classic complement pathway [19]. It has been proven that HBD-2 is widely expressed in the respiratory epithelium during various diseases, including pneumonia, and even serves a diagnostic purpose through evaluating the severity of it [21,22]. Other diseases include chronic rhinosinusitis and tuberculosis [23,24].

Another interesting study found that HBD-2 also takes part in bone defect reconstruction through upregulation of collagen 1, osteopontin and osteocalcin synthesis in rat models, proving the possibly variable nature of the peptide [25]. In addition, another study showed that HBD-2 also facilitates wound healing through better granulation in tissue formation and skin appendage regeneration [26]. Both of these studies prove that HBD-2 is not only an antimicrobial peptide, but also a pro-proliferative molecule which accelerates tissue regeneration and modulation. However, the studies on how widely HBD-2 is seen in cartilage tissues are scarce. It has been proven that HBD-2 is upregulated in osteoarthritic cartilage and could potentially be associated with pathogenesis of it, but more studies must be conducted [27]. Another study showed that synovial membranes also produce HBD-2 in case of infection in septic arthritis [28].

Human beta defensin 3 (HBD-3) is an antimicrobial peptide which is highly expressed in mucosal surfaces throughout the body, including respiratory and gastrointestinal systems as well as skin and gingiva, in response to pathogen- and damage-associated molecular patterns (PAMPs/DAMPs). It has been previously shown that HBD-3, similarly to HBD-2, causes bacterial wall disruption which continues to cause further bacterial osmotic lysis [29,30]. A study conducted by Ferris et al. demonstrated that HBD-3 can be found in the skin, where it activates dendritic cells that prime naïve T cells and promote their release of interferon γ, promoting both innate and adaptive immune responses [31]. In contrast, HBD-3 also has anti-inflammatory properties, especially whilst working in synergy with cathelicidin (LL-37). One of the possible pathways is through neutralisation of LPS, blocking the inflammatory potential of it and thus lowering the release of pro-inflammatory mediators such as IL-6 [32]. It has also been previously shown that human papillomavirus can induce HBD-3 expression in cervical mucosa, and that HBD-3 has not only antimicrobial effects but also pro-oncogenic effects, leading to the development of cervical cancer due to activation of NF-κB signalling [33].

Human beta defensin 4 (HBD-4) is another cationic antimicrobial peptide of the defensin family which is widely expressed in the gastrointestinal system, such as in the stomach where it can be induced by H. pylori infections and non-infectious gastritis [34]. Another place of expression is skin, where HBD-4 release is upregulated by bacterial infections such as Pseudomonas aeruginosa [35]. Other expression sites include gingival epithelium and dental pulp [36,37]. It has also been shown that HBD-4 is expressed in chondrocytes from articular cartilage, both in healthy subjects and subjects with osteoarthritis [38]. Just as with HBD-2 and HBD-3, this peptide shows antimicrobial activity through increased production of IL-6, IL-10, IFN-γ-inducible protein (IP-10), monocyte chemoattractant protein-1 (MCP-1) and macrophage inflammatory protein 3α (MIP-3α) [39].

Interleukin 10 (IL-10) is a cytokine that has pro- and anti-inflammatory properties. It is mainly produced by immune cells, including monocytes and B cells. Its functions include promotion of B cell survival, leading to prolonged and increased antibody production [40]. IL-10 also inhibits other cytokines which can be found in inflammatory sites, such as IL-8, IL-1β and TNFα, and lowers monocyte MHC molecule expression to downregulate the excessive inflammation [41]. Moreover, IL-10 has been found to normalize proteoglycan synthesis in blood-induced joint damage cases [17]. IL-10 also inhibits NOS2 and MMP-3 expression, thus showing another chondroprotective effect [18].

Human cathelicidin (LL-37) is an antimicrobial peptide expressed by epithelial cells of organs that have direct contact with the environment, such as airways, intestines and skin, which has a wide range of activity against gram-positive and gram-negative bacteria, viruses and fungi. One of the mechanisms is through membrane disruption, either using pressure (Carpet model), incorporation (barrel-stave model) or pore formation (toroidal pore model). Cathelicidins can also penetrate some cells and degrade necessary molecules related to that specific cell’s survival, as well as neutralize bacterial endotoxins. Furthermore, they also have immunomodulatory functions, including induction of neutrophil migration and chemotaxis, modulation of T cell differentiation, as well as modulation of macrophage and keratinocyte response to pathogens, thus amplifying IFN-1 response to viral RNA [29,42]. LL-37 is needed for angiogenesis and wound healing as it can stimulate fibroblasts and epithelial cells to proliferate in order to repair damage, though these are the same properties that are pro-carcinogenic when not being controlled properly in, for example, ovarian and breast cancers [43,44,45,46]. However, LL-37 also has an anti-carcinogenic effect as it kills cancer cells through mediation of natural killer cells, which only shows its dual properties in various processes in the human body [47].

As there is a lack of intercorrelations that have been studied between the distribution and appearance of different GAGs and hyaline cartilage protective tissue factors, the aim of this research was to evaluate the appearance of glycosaminoglycans as well as the appearance of specific antimicrobial defence molecules and cytokines in healthy tracheal hyaline cartilage tissues of different tracheal levels.

## 2. Materials and Methods

### 2.1. Material Characteristics of Subjects

The research was conducted in accordance with the Helsinki declaration at the Riga Stradins University Institute of Anatomy and Anthropology in Latvia. The study was approved by the Ethical committee at Riga Stradins University on the 20th of October 2021 (Project Nr. 22-2/471/2021). Tissue material was collected from the Institute of Anatomy and Anthropology archive. A total of 10 tracheal tissue samples were obtained from healthy men aged 30 to 60 years containing the complete tracheal wall including hyaline cartilage. We took the first three tracheal cartilage rings, fixed the side and then blindly chose 5 × 5 mm samples from 3 specific places named A, B and C, where A is left ring side, B is middle ring side and C is right ring side. To exclude any factors that could interfere with the results, patients were selected by inclusion and exclusion criteria. Inclusion criteria were male gender, age from 30 to 60 years and that the obtained tissue sample contained the whole tracheal wall, including hyaline cartilage, without macro- and microscopic pathological changes. Exclusion criteria were findings of any pathology in the tracheal tissue material.

### 2.2. Histochemical Research

The tissue samples were fixated for 24 h in a mixture of 2% formaldehyde and 0.2% picric acid in 0.1 M phosphate buffer that had a pH of 7.2. Tissues were then rinsed in Tyrode buffer that contained 10% saccharose for 12 h, and afterwards they were embedded into paraffin and later cut in 5 µm sections. Before coloring, the tissues were deparaffinized and rehydrated. For the general morphological evaluation of the wall of the trachea, routine staining with haematoxylin and eosin was performed. To evaluate glycosaminoglycan content in tracheal hyaline cartilage, the tissues were processed with Periodic acid–Schiff (PAS) and Bismarck brown staining.

For the PAS stain, the slides were placed into 0.5% periodic acid for 10 min, then stained with Schiff’s reagent for 20 min, followed by removal of excess reagent with potassium metabisulphite and counterstaining with Mayer’s hemalum solution for 3 min. The tissues were rinsed with distilled water after all steps, except the last one where tap water was used. The tissues were then dehydrated in alcohol, transferred into xylene and coverslipped [48,49,50].

Bismarck brown staining was performed by applying Bismarck brown reagent (0.5 g Bismarck brown stain, 80 mL 96% alcohol and 20 mL 1% HCl) for 2 h to tissues at room temperature followed by tissue differentiation in 3 rounds with 70% ethanol. The tissues then were counterstained with Mayer’s haematoxylin, rinsed, dehydrated, cleared in xylene and locked into histological glue [51].

For Masson trichrome staining (Bio-Optica Milano S.p.A., Milan, Italy), the tissues were brought into distilled water and then processed with Wiegert’s iron haematoxylin solutions A and B for 10 min and picric acid alcohol solution for 4 min. Ponceau acid fuchsin according to Mallory was then applied for 4 min and phosphomolybdic acid solution was added afterwards for 10 min and Masson aniline blue for 5 min. Lastly, tissues were rinsed, dehydrated in ascending alcohols, cleared in xylene and mounted [52].

### 2.3. Immunohistochemical (IHC) Analysis

IHC labelling was achieved with the use of the standard Biotin—Streptavidin method to detect: HBD-2 (ab63982, working dilution 1:500, Abcam, Cambridge, UK), HBD-3 (orb183268, working dilution 1:100, Biorbyt Limited, Cambridge, UK), HBD-4 (ab70215, working dilution 1:100, Abcam, Cambridge, UK), IL-10 (orb100193, working dilution 1:600, Biorbyt Limited, Cambridge, UK), and LL-37 (orb88370, working dilution 1:100, Biorbyt LLC, St Louis, MO, USA) [53,54].

The sample slides were analysed by light microscopy using non-parametric evaluation, which included grading of positively stained tracheal hyaline cartilage cells in the visual field. The results were labelled as follows: 0—no positive cells; 00/+—scant number of positive cells; 0/+—occasional positive cells; +—few positive cells; +/++—few to moderate number of positive cells; ++—moderate number of positive cells; ++/+++—moderate to numerous positive cells; +++—numerous positive cells; +++/++++—numerous to abundant positive cells; ++++—abundant number of positive cells [55].

The extracellular matrix was also analysed in Bismarck brown and PAS staining for neutral and acidic glycosaminoglycans, and the results were labelled accordingly: -—no positive ground substance; ±—partially stained ground substance; +—stained ground substance.

### 2.4. Statistical Analysis

The obtained data processing was performed with IBM SPSS software version 27.0 (IBM company, North Castle, Armonk, NY, USA). To evaluate correlation between the GAGs and cartilage protective tissue factors in this study we used Spearman’s rank correlation coefficient, where R < 0.2 indicated a very weak correlation, R = 0.2–0.4 was assumed as a weak correlation, R = 0.4–0.6 a moderate correlation, R = 0.6–0.8 a strong correlation and R = 0.8–1 a very strong correlation.

## 3. Results

**Figure 1:**
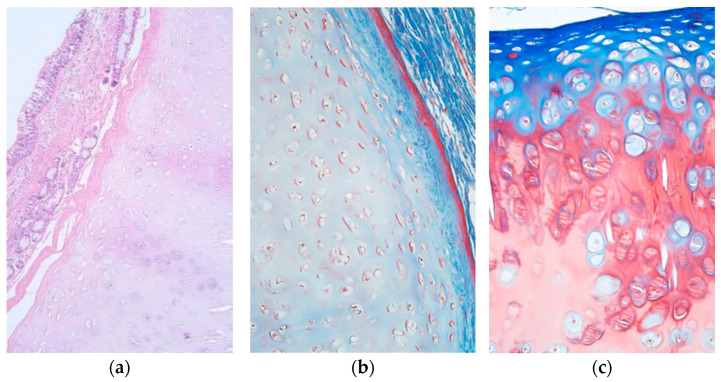
(**a**–**c**) Micrographs of tracheal structures in healthy subjects. (**a**) Unchanged tracheal wall containing pseudostratified ciliated epithelium, seromucous glands and tracheal hyaline cartilage. Haematoxylin and eosin, ×100. (**b**) Tracheal hyaline cartilage without signs of ossification. Masson’s trichrome, ×200. (**c**) Note the ossification of tracheal hyaline cartilage. Masson’s trichrome, ×200.

Tissue samples that were obtained in this study contained the whole tracheal wall, including ciliated pseudostratified epithelium, mucosal connective tissue, seromucous glands, tracheal smooth muscle, as well as hyaline cartilage without any inflammatory changes. Masson’s trichrome staining revealed ossification of the cartilage in some of the patient samples (Figure 1a–c).

**Figure 2:**
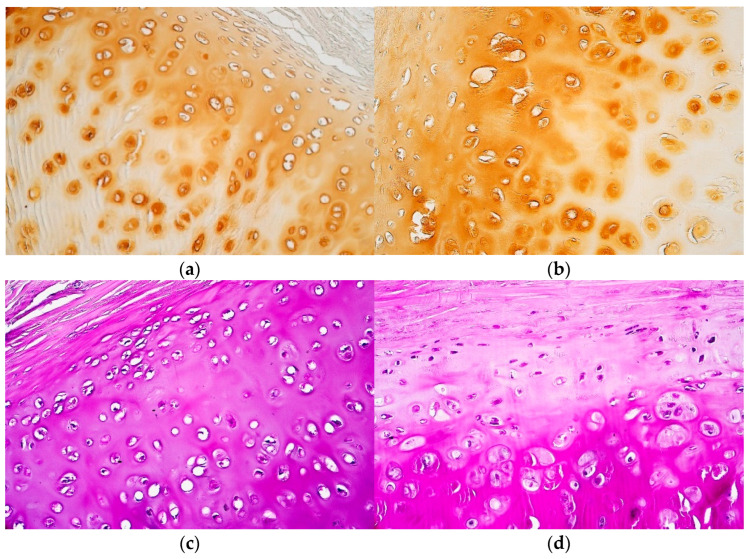
(**a**–**d**) Micrographs of tracheal hyaline cartilage stained with Bismarck brown and PAS: (**a**) Numerous positive Bismarck brown cells throughout all the zones of tracheal hyaline cartilage. Note the Bismarck brown positive extracellular matrix of proliferation and growth zones. Bismarck brown, ×200; (**b**) Note numerous positive cells across all hyaline cartilage zones together with the positive extracellular matrix in proliferation and growth zones. Bismarck brown, ×200; (**c**) Positive extracellular matrix throughout the whole hyaline cartilage. Periodic acid–Schiff, ×200; (**d**) Numerous positive cells across all of the hyaline cartilage zones. Note the intensively positive extracellular matrix in the mature cell zone. Periodic acid–Schiff, ×200.

Bismarck brown staining was positive in the extracellular matrix of proliferation and growth zones in all patient tissue samples; however, only 3 out of 27 were positive and 6 were partially positive in the mature cell zone. This staining also marked numerous positive cells in all of the cartilage zones (Table 1 and Table 2, Figure 2a,b).

Periodic acid–Schiff (PAS) staining was positive in the proliferation zone of 17 samples and 8 were also partially positive. Moreover, PAS stained 17 positive growth zones, while an additional 10 were partially positive. Lastly, almost all mature cell zones were positive, with the exception of two samples that were only partially positive. All of the tissue samples contained numerous PAS positive cells in proliferation, growth and mature cell zones (Table 1 and Table 2, Figure 2c,d).

**Figure 3:**
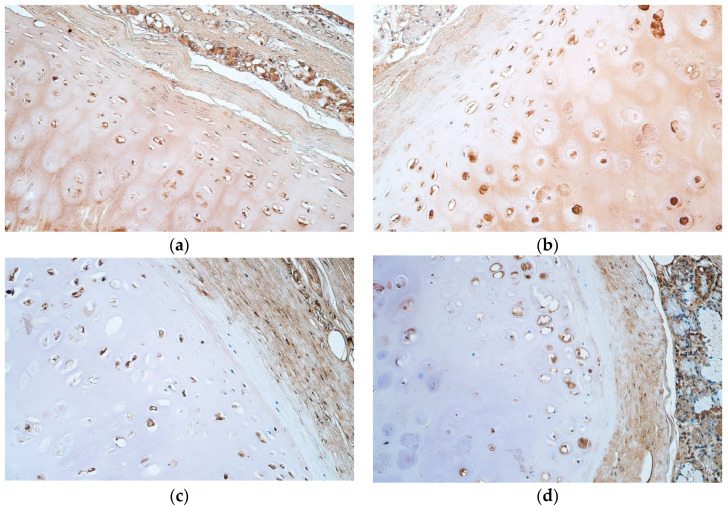

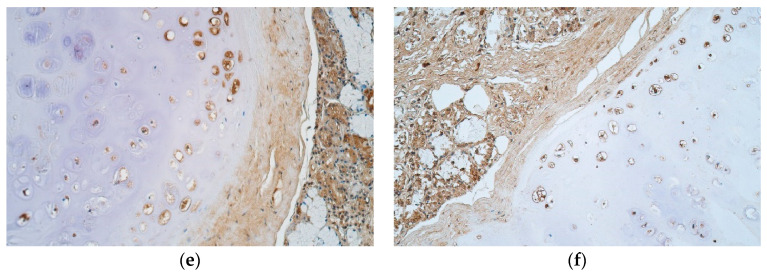
(**a**–**f**) Immunohistochemical micrographs of tracheal hyaline cartilage: (**a**) Moderate number of HBD-2 positive cells across proliferation and growth zones. HBD-2 IMH, ×200; (**b**) HBD-2 positive cells in moderate number across the proliferation zone with numerous positive cells in the growth zone. HBD-2 IMH, ×200; (**c**) Moderate number of HBD-3 positive cells in the growth zone with only few positive cells in the mature cell zone. HBD-3 IMH, ×200; (**d**) Note the moderate amount of HBD-3 positive cells in the proliferation and growth zones with only few positive cells in the mature cell zone. HBD-3 IMH, ×200; (**e**) Moderate amount of HBD-4 positive cells in the proliferation and growth zones with only few positive cells in the mature cell zone. HBD-4 IMH, ×200; (**f**) HBD-4 positive cells in moderate number throughout proliferation and growth zones with few positive cells in the mature cell zone. HBD-4 IMH, ×200.

A moderate number of HBD-2 positive cells were seen in the proliferation and mature cell zones, but a moderate to numerous amount of cells was found in the growth zone of the tracheal hyaline cartilage (Table 3, Figure 3a,b).

HBD-3 positive cells were few to moderate in number in the proliferation zone, moderate in the growth zone, and few in the mature cell zone of the tracheal hyaline cartilage (Table 3, Figure 3c,d).

HBD-4 was few to moderate in number for positive cells in the proliferation zone, moderate in number for positive cells in the growth zone and few in number for the mature cell zone (Table 3, Figure 3e,f).

**Figure 4:**
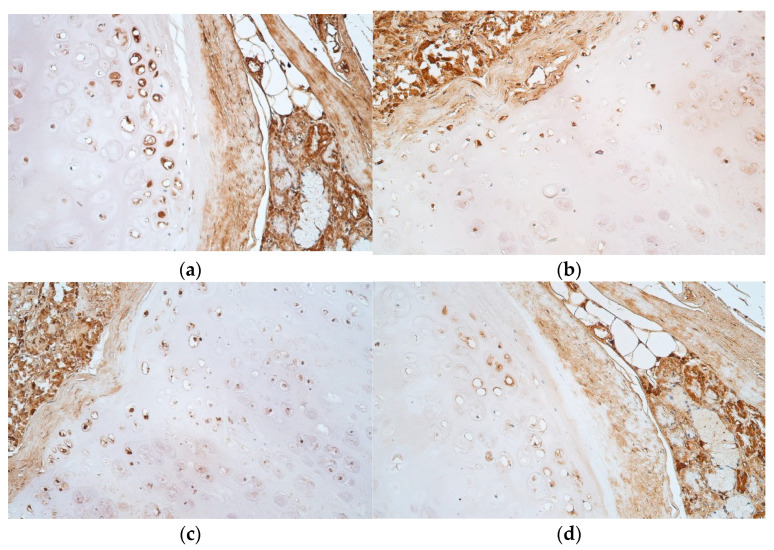
(**a**–**d**) Immunohistochemical micrographs of tracheal hyaline cartilage: (**a**) Moderate number of IL-10 positive cells in proliferation and growth zones. IL-10 IMH, ×200; (**b**) A moderate number of IL-10 positive cells across the proliferation zone. IL-10 IMH, ×200; (**c**) Moderate number of LL-37 positive cells across proliferation and growth zones. LL-37 IMH, ×200; (**d**) Note the moderate number of LL-37 positive cells in the growth zone. LL-37 IMH, ×200.

IL-10 positive cells were seen more in the growth zone, where they were moderate in number. The proliferation zone contained few to moderate IL-10 positive cells and the mature cell zone had mostly few, although the results varied from no positive cells to a moderate number of them (Table 4, Figure 4a,b).

LL-37 marked a moderate number of positive cells both in proliferation and growth zones, whilst the mature cell zone contained occasional positive cells (Table 4, Figure 4c,d).

Spearman’s rank correlation coefficient revealed a strong positive correlation in the healthy tracheal hyaline cartilage between HBD-3 and IL-10 and HBD-4 and LL-37. Moderate positive correlation was detected between HBD-3 and HBD-4, HBD-3 and LL-37, as well as between IL-10 and LL-37. A moderate negative correlation was observed between PAS and LL-37 (Table 5).

## 4. Discussion

Hyaline cartilage is a very important part of the trachea, as one of its functions is providing structural scaffolding for the organ in order to keep the airways open and protect them from mechanical damage [1]. The main components of the hyaline cartilage of the trachea, which supports this function, are glycosaminoglycans that are synthesized by cartilaginous cells and secreted in the extracellular matrix, where they additionally support intercellular signalling and nutrition of the cells [11]. There are two main types of GAGs that can be assessed: acidic (PAS negative and Bismarck brown positive) and neutral (PAS staining positive) [56,57]. Previous studies have shown that cartilage contains chondroitin sulphate, which is a key component of water retention and cushioning of the cartilage, keratan sulphate, which is responsible for ion gradient control and stimulation of cell differentiation and proliferation, and hyaluronic acid, which provides hydration of cartilage in order to provide similar lubrication and protection as chondroitin sulphate while also stimulating the flow of nutrients and waste products to and from cartilaginous cells [11,12,13,14]. In this study, it was found that neutral GAGs can be found in the cartilage cells, both chondroblasts and chondrocytes, as well as in the extracellular matrix of all of the tracheal hyaline cartilage zones, which means that they are the key components of providing mechanical structural integrity and intercellular signalling in hyaline cartilage. Moreover, acidic glycosaminoglycans are also found in cartilaginous cells across all zones, though our study shows that in the extracellular matrix they are mostly present in proliferation and growth zones of the hyaline cartilage. Thus, the mature cell zone is mainly supported by neutral GAGs.

However, tracheal hyaline cartilage possesses not only the function of airway structural support and air conduction, but also serves as a defence structure against pathogens invading the organ [9]. All of the studied factors—HBD-2, HBD-3, HBD-4, LL-37 and IL-10—were found in mostly moderate number across the proliferation and growth zones and few positive cells were found in the mature cell zone, which means that the cells of the hyaline cartilage in the periphery are more active when it comes to synthesis of antimicrobial molecules and cytokines. That could be explained by the fact that hyaline cartilage is an avascular structure and the source of nutrients and regulatory molecules is perichondrium, which is closest to the proliferation and growth zones and which can induce the expression of the molecules researched [9].

Defensins are small antimicrobial peptides that, through the innate immune system, protect the organism against different pathogens. They are found not only in the epithelium, but also in immune cells, such as monocytes and macrophages, and are secreted in response to cytokines, Toll-like receptor activation and microorganisms themselves [58]. Our data shows that they are apparent in hyaline cartilage even in normal physiologic conditions, which could be beneficial for providing quick and effective protection in case a pathogen does come into the contact with cartilaginous tissues of the trachea. Moreover, the trachea is not a sterile organ, as it encounters a variety of microorganisms that sometimes can induce immune reactions which could influence the epithelium and spread into deeper tissues as well [59].

HBD-2 is a defensin that has been widely described in association with respiratory tract diseases but not, however, in association with tracheal pathology specifically [21,22,23,24]. HBD-2 has multiple roles in immune processes, for example, it induces pore complex formation and lysis of various microorganisms [19]. Additionally, HBD-2 also induces cytokine gene upregulation and provides effective T cell and neutrophil leucocyte recruitment to the tissues when needed [60,61]. Interestingly, in contrast to that, HBD-2 also poses an anti-inflammatory characteristic, as it has been proven to inhibit the classic complement system in case of its activation, thus lowering the immune response to pathogens in some instances [19]. To support the complex nature of this defensin, HBD-2 also has a pro-proliferative function, as it takes a part in bone defect reconstruction and facilitates wound healing [25,26]. HBD-2 in tracheal hyaline cartilage possibly has not only the role of providing immunity, but could also potentially work as an anti-inflammatory and pro-proliferative molecule, both of which are characteristics that are beneficial in sustaining its structural integrity. Thus, we speculate that HBD-2 also has a multifactorial role in our patients. Additionally, it is also very important to note that HBD-2 is elevated in chronic lung allograft rejection cases when, after the transplantation, bronchiolitis obliterans syndrome develops due to donor specific antibody formation and immune reaction. Thus, HBD-2 could possibly be used as a predictive marker in early transplant rejection [60].

HBD-3 is a member of the beta defensin family which is positively charged and has some of the broadest and strongest antimicrobial activity [62]. Similar to HBD-2, HBD-3 also induces microorganism wall destruction and lysis, as well as inducing chemokine production (IL-1α, IL-6 and TNFα) and activation of other immune cells, such as macrophages, dendritic cells and T cells [29,30,31,61]. Additionally, HBD-3 has also been proven to have anti-inflammatory properties such as neutralisation of bacterial LPS and lowered release of IL-6 and TNF-α [32,62]. HBD-3 also facilitates wound healing through promotion of epithelial cell migration in rat intestines [63]. Thus, the functions of HBD-3 do have some similarity to HBD-2 effects; however, due to cationic properties and broad-spectrum antimicrobial activity, we suspect that HBD-3 could be most beneficial out of all defensins for protection of the trachea against pathogens.

Lastly, HBD-4, a potent member of the defensin family with a scarce distribution more often found in cases of gastrointestinal tract and skin pathologies, was also found in the tracheal hyaline cartilage of our patient samples as HBD-2 and HBD-3 were, with the highest positive cell count in the growth zone [34,35]. However, HBD-4 has its own distinct properties as, in contrast to other defensins, its promoter region does not contain a binding site of inflammatory mediator NF-κB, which is one of the main regulators of innate immunity [64]. Moreover, HBD-4 has been proven to have an important role in lower respiratory tract innate immunity and, together with HBD-2, it has been researched in correlation with COVID-19 infections. It has been proven that, dissimilar to HBD-2 which was downregulated in patient cases, HBD-4 was highly upregulated, which shows that its effects might differ from other defensins even more so [65,66]. Although studies about HBD-4 expression in cartilage have been very scarce, this molecule has been shown to be present in articular cartilage of the knee in healthy humans, which further supports our idea that this molecule is very important in promoting baseline immunity of tracheal hyaline cartilage as well. Interestingly, out of all defensins, HBD-4 has been shown to decrease in osteoarthritic knee cartilage which has been treated with autologous cell transplantation, serving as a marker of successful treatment. Thus it could also possibly have a potential role in the investigation of tracheal transplantation in future studies [38].

Our study also revealed that LL-37, a potent antimicrobial molecule, was present in tracheal hyaline cartilage, especially in proliferation and growth zones indicating its protective role there. LL-37 is expressed mostly in epitheliocytes of organs that have lumens and that are in a direct contact with the environment and microorganisms, such as the gastrointestinal and respiratory tract. One of the antimicrobial effects that it possesses is the ability to disrupt membranes through various pathways, most importantly through pore formation. Furthermore, it can induce not only chemotaxis of neutrophils and monocytes, but also T cell differentiation and synthesis of IL-8, IL-6, IL-1β, and TNF-α [44,67]. In the epithelium itself, it has been proven to increase IFN-1 secretion in response to viral pathogens and it can thus be pointed out that LL-37 plays a great role in innate and adaptive immune processes [29]. LL-37 is also needed in wound healing, as it promotes epitheliocyte and fibroblast proliferation and synthesis of IL-6 and IL-1 [44,67]. As our data shows that LL-37 is present in human tracheal hyaline cartilage, all of the previously mentioned functions could have a role in protection of the trachea. Interestingly, elevated levels of LL-37, as is the case with HBD-2, are also associated with bronchiolitis obliterans syndrome and pathogenesis of transplant rejection, supporting the fact that this molecule could also be a beneficial molecular target for tracheal transplantation rejection case investigation [68].

As with other previously mentioned antimicrobial peptides, cytokine IL-10 was also found throughout all hyaline cartilage zones, though with the lowest number of positive cells in the mature cell zone. Thus, it proves that this interleukin is also needed for baseline immunity of the trachea. However, IL-10 is a cytokine which possesses very well-known dual activity as it has both pro-inflammatory and anti-inflammatory properties, out of which one of the most important effects is the downregulation of other cytokines, such as IFN-γ, IL-6, IL-1β, IL-15, IL-23 and TNFα [40,41,69,70]. Moreover, IL-10 also exhibits other effects on the cartilage, more specifically on extracellular matrix production and degradation. To provide an example, a study made by Müller et al. showed that TNFα, which is responsible for aggrecan expression downregulation and MMP-13 upregulation in human articular chondrocytes, is inhibited by IL-10 [71]. Moreover, IL-10 has been found to normalize proteoglycan synthesis in blood-induced joint damage cases [17]. IL-10 also inhibits NOS2 and MMP-3 expression, thus resulting in the excessive degradation of the extracellular matrix slowing down [18]. Additionally, a study performed by Waly et al. in 2017 showed that glucosamine treatment in mice has a chondroprotective effect by increasing IL-10 and decreasing TGF-β1 levels [72]. Thus, it could be possible that IL-10 does not only have immunomodulatory properties, as it is possibly a very important regulator of extracellular matrix content in tracheal hyaline cartilage.

Our study also revealed correlations between the studied factors. First of all, a strong positive correlation was noted between HBD-3 and IL-10; however, it has been proven that these molecules work individually, not synergistically [63]. Moreover, there was a moderate positive correlation between LL-37 and IL-10. HBD-3 and LL-37 both have been proven to be able to induce expression of IL-1 and TNFα, both of which can also induce IL-10 expression. Thus, it could be that both of these correlations can be explained by the pathway, in which HBD-3 and LL-37 induces TNFα and IL-1 synthesis which in turn stimulates IL-10 expression [61,67,71,73].

There was also a strong positive correlation between HBD-4 and LL-37 and a moderate positive correlation between HBD-3 and LL-37. The synergistic activity of HBDs and LL-37 has been noted in previous studies, such as in association with activation of IL-18 secretion in primary human keratinocytes, possibly through caspase-1 independent mechanisms [74]. Additionally, HBD-3 and HBD-4, together with LL-37, have been proven to induce IL-31 secretion from human mast cells, as well as stimulate the release of IL-2, IL-4, IL-6, nerve growth factor, PGE2 and leukotriene C4 [75]. This synergistic activity could be explained by the fact that LL-37 is known to induce different mediator secretion, for example TNFα, which in return can upregulate HBD-3 and HBD-4 [37,67,76]. The similar induction pathways of HBD-3 and HBD-4 could also explain the moderate positive correlation between these factors which was also observed in our study.

Finally, this study also shows a moderate negative correlation between PAS stained neutral glycosaminoglycans and LL-37. This finding can be supported by the fact that LL-37 is known to be inhibited by GAGs due to them binding to each other, which causes lower microorganism binding to the LL-37 in vitro [77]. Furthermore, a study made by Bergsson et al. shows that LL-37 antimicrobial activity is also inhibited in cystic fibrosis patients due to glycosaminoglycan activity, and that this feature can be improved by the administration of hypertonic saline [78]. However, this also can serve as a protective factor, as LL-37 can induce acute inflammation and apoptosis in urothelial cells and these effects are significantly lowered by administration of glycosaminoglycans into the tissues [79]. Previously mentioned LL-37 inhibition by GAGs could also have a benefit in tracheal hyaline cartilage by downregulating LL-37 effects to avoid excessive immune response into the tissues.

Interestingly, tracheal cartilage shares similarities with hyaline cartilage found in other places. For example, HBD-4 has been proven to be weakly expressed in healthy knee cartilage; however, HBD-2 and HBD-3 have been proven to be absent. Moreover, all of the human beta defensins included in this study are increased in osteoarthritic knee cartilage, which proves that these factors could play a role in its pathogenesis [27,38,80]. Nasal hyaline cartilage, which has been previously studied in relation to pathologies such as cleft palate and lip or surgical infection, contains not only factors such as TGF-β1, but also HBD-2 and HBD-3, which shows that there could be more similarities expected [81,82]. Human beta defensins are a group of antimicrobial molecules with tissue regenerative properties, e.g., HBD-2 is involved in bone defect reconstruction and also induces wound healing by stimulating granulation tissue to form and accelerating skin appendage regeneration [25,26]. Furthermore, nasal chondrocytes have been used in regenerative medicine in order to repair knee cartilage defects and we thus suspect that tracheal hyaline cartilage also could possess some beneficial properties [83,84].

Our study has some limitations. One of them is the small number of patient samples, especially for the three points of each tracheal hyaline cartilage ring, which makes it impossible to precisely evaluate glycosaminoglycan distribution. However, we inspected the tissues of healthy adults and, due to ethical concerns, they are very hard to obtain. This is also the reason why it is difficult to evaluate changes in the studied factors throughout different age groups, as in our study there were no specific differences noted in subjects aged from 30 to 60; this does not exclude the possibility that in younger or older subjects there could be possible changes and more patient samples are therefore needed in order to investigate this. Moreover, we used immunohistochemistry to evaluate studied factors in the tissue samples. If we had used other methods, such as ELISA or identification of genes involved in factor production in normal physiological conditions, we might have obtained more precise results. Thus, in future research we are planning to continue this study and correct some of these issues.

## 5. Conclusions

The extracellular matrix of tracheal hyaline cartilage, which is composed of both neutral and acidic glycosaminoglycans that are also found in numerous positive cells across all of the cartilage zones, provides structural scaffolding of trachea and supports cell nutrition and signalling throughout all cartilaginous zones; however, the persistence of nGAGs in the mature cell zone correlates more to the aging of the same cartilage.

The hyaline cartilage of the trachea is also an important defence structure which contains a moderate number of antimicrobial defence protein and cytokine immunoreactive cells in normophysiological conditions. The appearance of human beta defensin 2, 3 and 4 as well as LL-37 and IL-10 mainly in proliferation and growth zone indicates these zones as the more active ones in the synthesis of these factors.

The correlations between the studied factors (especially between HBD-3 and IL-10, HBD-3 and LL-37, and nGAGs and LL-37) confirm that they are working in a synergistic manner in order to support tracheal baseline immunity and protect the organ from possible microorganism invasion.

## Figures and Tables

**Table 1:** Distribution and appearance of extracellular matrix positives for Bismarck brown and PAS staining in healthy tracheal hyaline cartilage.

Patient	Cartilage Nr.	Part of Cartilage	BB			PAS		
			PZ	GZ	MCZ	PZ	GZ	MCZ
1	1	A	+	+	-	±	±	+
	2	A	+	+	-	±	±	+
	3	A	+	+	-	+	+	+
		B	+	+	-	+	+	+
		C	+	+	-	-	±	+
2	1	A	+	+	±	+	+	+
		C	+	+	-	+	+	+
	3	A	+	+	±	+	+	+
3	1	A	+	+	-	-	±	+
	3	A	+	+	-	+	+	+
		B	+	+	±	+	+	+
4	1	A	+	+	±	+	+	+
		C	+	+	±	+	+	+
5	1	A	+	+	-	+	+	+
		B	+	+	-	+	+	+
	2	A	+	+	-	+	+	+
6	3	A	+	+	+	+	+	+
7	2	A	+	+	+	±	±	+
		B	+	+	+	+	+	±
8	1	A	+	+	-	±	±	±
		B	+	+	±	±	±	+
9	1	A	+	+	-	+	+	+
	2	A	+	+	-	±	±	+
		B	+	+	-	±	±	+
10	1	A	+	+	-	±	±	+
		B	+	+	-	+	+	+
		C	+	+	-	+	+	+
Median value			+	+	-	+	+	+

Abbreviations: A—left ring side; B—middle ring side; C—right ring side; PZ—proliferation zone; GZ—growth zone; MCZ—mature cell zone; BB—Bismarck brown staining; PAS—Periodic acid–Schiff staining; -—no positive ground substance; ±—partially stained ground substance; +— stained ground substance.

**Table 2:** Relative number of Bismarck brown and Periodic acid–Schiff positive cells in different tracheal hyaline cartilage zones.

Patient	Cartilage Nr.	Part of Cartilage	BB			PAS		
			PZ	GZ	MCZ	PZ	GZ	MCZ
1	1	A	+++	+++	+++	+	++	++++
	2	A	+++	+++	+++	+++	+++	++++
	3	A	+++	+++	++++	+++	+++	+++
		B	++	+++	+++	+++	+++	+++
		C	++/+++	+++	+++	+++	+++/++++	+++
2	1	A	+++	++++	++++	+++	+++	+++
		C	+++	++++	++++	++++	++++	++++
	3	A	++++	++++	++++	+++	+++	+++
3	1	A	++/+++	++++	+++	++	++	++/+++
	3	A	++/+++	+++	++/+++	++	++	++/+++
		B	+++/++++	++++	+++/++++	+++	+++	+++
4	1	A	+++	+++	+++	+++	+++	+++
		C	+++	+++	+++	+++	+++	+++
5	1	A	+++	+++	+++	+++	+++	+++/++++
		B	++/+++	+++	++++	+++	++++	++++
	2	A	++/+++	+++	+++	+++	+++	++++
6	3	A	+++	+++	+++	+++	+++	+++
7	2	A	+++	+++	+++	+++	+++	+++
		B	++	++	++	+++	+++	+++
8	1	A	+++	+++	+++/++++	+++	+++	+++
		B	+++	+++	+++	+++	+++	+++
9	1	A	+++	+++	+++	++	+++	+++
	2	A	++	+++	+++	+++	+++	+++
		B	+++	++++	++++	++	++	+++
10	1	A	+++	+++	+++	++	+++	+++
		B	+++	+++/++++	+++	+++	+++	+++
		C	+++	+++	+++	+++	+++	+++
Median value			+++	+++	+++	+++	+++	+++

Abbreviations: A—left ring side; B—middle ring side; C—right ring side; PZ—proliferation zone; GZ—growth zone; MCZ—mature cell zone; BB—Bismarck brown staining; PAS—Periodic acid–Schiff staining; +—few positive cells; ++—moderate number of positive cells; ++/+++—moderate to numerous positive cells; +++—numerous positive cells in the visual field; +++/++++—numerous to abundant positive cells; ++++—abundant number of positive cells.

**Table 3:** Relative number of human beta defensin positive cells in different tracheal hyaline cartilage zones.

Patient	Cartilage Nr.	Part of Cartilage	HBD-2			HBD-3			HBD-4		
			PZ	GZ	MCZ	PZ	GZ	MCZ	PZ	GZ	MCZ
1	1	A	+/++	++	++	+/++	++	0/+	++	++	+
	2	A	++/+++	+++/++++	++	++	++	+	++	++	+
	3	A	++	++/+++	++	+	++	0/+	+/++	++	+
		B	++	++	++	++	++	+	+	+/++	0/+
		C	++/+++	++/+++	++	+/++	++	0/+	0/+	+/++	0/+
2	1	A	++	++/+++	++/+++	++/+++	+++/++++	++	+	++	++
		C	++	++	++	++	+++	+/++	+	+/++	+/++
	3	A	++	++/+++	++/+++	+/++	+++	+/++	+/++	++	++
3	1	A	++	++/+++	++	+/++	+/++	0/+	+/++	++	0/+
	3	A	++/+++	++/+++	++	++	++	+	++	++	+
		B	++	+++	++/+++	++	++	0/+	+/++	++	+
4	1	A	++	++	++	+/++	++	+	+	+/++	+
		C	++	++	++	+	+/++	+	+	+/++	+
5	1	A	N	N	N	+	+	0/+	+/++	++	+
		B	++	++/+++	++	+/++	++	+	+/++	++	+
	2	A	+/++	++	+/++	0/+	+	0	+/++	+/++	+
6	3	A	++/+++	++/+++	++	+	+	0	+/++	+/++	0/+
7	2	A	+++	+++/++++	+++	+/++	++	+	++	++	+/++
		B	+++	+++	+++	++	++	+	+/++	++	+/++
8	1	A	++/+++	++/+++	++	+	+/++	0	+/++	+/++	+
		B	+/++	++	+/++	+	+/++	+	+/++	+/++	0/+
9	1	A	++	++	+/++	++	++/+++	+	++	++/+++	+
	2	A	++	++	+/++	++	++	+	++	++	+
		B	++	++/+++	++	N	N	N	++	++/+++	+
10	1	A	+/++	++	++	+/++	+/++	0/+	+	+	0/+
		B	+/++	++	++	+/++	+/++	0/+	+/++	+	0/+
		C	++	++/+++	++	++	+	0/+	++	+/++	0/+
Median value			++	++/+++	++	+/++	++	+	+/++	++	+

Abbreviations: A—left ring side; B—middle ring side; C—right ring side; PZ—proliferation zone; GZ—growth zone; MCZ—mature cell zone; HBD2—human beta defensin 2; HBD3—human beta defensin 3; HBD4—human beta defensin 4; N—no tissue sample; 0—no positive cells; 0/+—occasional positive cells; +—few positive cells; +/++—few to moderate number of positive cells; ++—moderate number of positive cells; ++/+++—moderate to numerous positive cells; +++—numerous positive cells in the visual field; +++/++++—numerous to abundant positive cells.

**Table 4:** Relative number of interleukin 10 and human cathelicidin positive cells in different tracheal hyaline cartilage zones.

Patient	Cartilage Nr.	Part of Cartilage	IL-10			LL-37		
			PZ	GZ	MCZ	PZ	GZ	MCZ
1	1	A	+/++	++	0	++	++	+
	2	A	++	+	0/+	++	++	00/+
	3	A	+/++	++	+/++	++	++	0
		B	+	++	+	+/++	++	00/+
		C	+	++	+/++	+	+/++	0
2	1	A	+/++	++	++	+/++	++/+++	++
		A	+/++	++	++	+/++	+/++	+
	3	B	+	++	+/++	++	+++	+/++
3	1	A	+/++	+	0/+	++	++	00/+
	3	A	+	+	0	++	++	0/+
		B	+/++	++	+	++	++/+++	0/+
4	1	A	+/++	++	+	++	++	+
		C	+/++	++	+	+/++	+/++	+
5	1	A	N	N	N	N	N	N
		B	+/++	+/++	0/+	+	+	00/+
	2	A	+	+	0/+	+	+	00/+
6	3	A	+	+	0	+/++	+/++	0
7	2	A	+/++	++	+	++/+++	++	+/++
		B	+	++	+	+/++	+/++	0/+
8	1	A	+	+/++	0/+	+	+	0
		B	+	+	0/+	+/++	+/++	00/+
9	1	A	++	++	+	++/+++	+++	0
	2	A	++	++	+/++	++	++	0/+
		B	NS	NS	NS	++/+++	+++	0
10	1	A	+	+	0/+	+/++	+/++	0
		B	+	+/++	+	++	++	0/+
		C	+/++	+	0/+	++	+/++	00/+
Median value			+/++	++	+	++	++	0/+

Abbreviations: A—left ring side; B—middle ring side; C—right ring side; PZ—proliferation zone; GZ—growth zone; MCZ—mature cell zone; IL-10—interleukin 10; LL-37—human cathelicidin; N—no tissue sample; 0—no positive cells; 00/+—a scant number of positive cells; 0/+—occasional positive cells; +—few positive cells; +/++—few to moderate number of positive cells; ++—moderate number of positive cells; ++/+++—moderate to numerous positive cells; +++—numerous positive cells in the visual field.

**Table 5:** Spearman’s rank correlation coefficient revealing correlations between different factors in tracheal hyaline cartilage.

Factor 1	Factor 2	R	*p*-Value
A strong correlation (0.6–0.8)			
HBD3	IL-10	0.623	0.001
HBD4	LL-37	0.639	<0.001
A moderate correlation (0.4–0.6)			
HBD3	HBD4	0.534	0.005
HBD3	LL-37	0.593	0.002
IL-10	LL-37	0.521	0.008
PAS	LL-37	−0.410	0.037

Abbreviations: HBD3—human beta defensin 3; HBD4—human beta defensin 4; IL-10—interleukin 10; LL-37—human cathelicidin; PAS—Periodic acid–Schiff.

## Data Availability

All datasets used/analysed in the present study are presented in the result sections of the manuscript.
